# Quality by Design Approach for Hot-Melt Extrusion Coupled Fused Deposition Modeling (HME-FDM) 3D Printing: A Systematic Review

**DOI:** 10.3390/pharmaceutics18050569

**Published:** 2026-05-02

**Authors:** Petra Arany, Ádám Papp, Dániel Nemes, Pálma Fehér, Zoltán Ujhelyi, Ildikó Bácskay

**Affiliations:** 1 Department of Pharmaceutical Technology, Faculty of Pharmacy, University of Debrecen, 1 Rex Ferenc Utca, H-4002 Debrecen, Hungary; nemes.daniel@pharm.unideb.hu (D.N.); feher.palma@pharm.unideb.hu (P.F.); bacskay.ildiko@pharm.unideb.hu (I.B.); 2 Doctoral School of Pharmaceutical Sciences, University of Debrecen, H-4032 Debrecen, Hungary; papp.adam@med.unideb.hu; 3 University Pharmacy, Health Care Service Units, University of Debrecen Clinical Centre, University of Debrecen, 98 Nagyerdei Körút, H-4032 Debrecen, Hungary; 4 Department of Pharmaceutical Industry and Pharmaceutical Technology, Faculty of Pharmacy, University of Debrecen, 1 Rex Ferenc Utca, H-4002 Debrecen, Hungary; ujhelyi.zoltan@pharm.unideb.hu

**Keywords:** 3D printing, fused deposition modeling, hot-melt extrusion, quality by design, critical printing parameters, process analytical technologies

## Abstract

**Background:** Fused deposition modeling (FDM) is one of the most well-known and often published methods for 3D-printed drug delivery systems. In early scientific reports, the active pharmaceutical ingredients were added by soaking, but later, a new milestone was established, after researchers started to manufacture their own filaments by hot-melt extrusion (HME). The number of publications covering this method has multiplied in the last decade, a wide range of natural and synthetic polymers have been tested with versatile active pharmaceutical ingredient components, and various printing parameters and their effects have been investigated. **Objectives:** In this review, we aim to synthesize how the available quality by design approaches and the scientific results established so far can facilitate the creation of a guideline for appropriate quality production of HME-FDM 3D-printed pharmaceuticals. **Methods:** Based on PRISMA 2020 guidelines, a systematic search of relevant publications from 2015 to 2025 was carried out using the PubMed database. Twenty-six articles were included, based on number of monitored parameters and methodological description. Reporting of important quality processes and material parameters was assessed. **Results:** HME, the FDM, and analytical testing experiences were compared and collected into three tables from the selected publications. In two different sections, the pharmacopeial dosage-form tests and the involvement of process analytical technologies (PAT) were also analyzed. We found that reporting of influential parameters is heterogenous, and lack of robust reporting schemes limits the development of QbD approaches. **Conclusions:** Regarding the data, trends were synthetized, and a guideline was created which is limited by inconsistent parameter reporting.

## 1. Introduction

Fused deposition modeling (FDM) is one of the most frequently used 3D printing methods, a technique which is based on the deposition of melted layers of thermoplastic materials followed by solidification at room temperature. Such a process holds huge potential for the manufacturing of pharmaceutical products and is currently under extensive investigation [1,2]. It offers several undeniable advantages such as cost-effectiveness, design-flexibility without the need for changing tools, the potential to fabricate complex geometries, and enabling on-demand production of customized components with reduced material waste and lead time compared to traditional systems [3]. Nevertheless, several possible limitations need to be considered, like the limited resolution and surface quality, anisotropic length, and its slowness compared to other 3D printing methods [4]. In case of the pharmaceutical aspect the greatest challenge is the ventilation and the high printing temperature which can lead to the degradation of the active pharmaceutical ingredient (API) and decomposition [5].

In early research, APIs were incorporated into the excipients by soaking: the printable polymer filaments were immersed in a solution or dispersion containing the API. Usually a maximum of 0.06% *w*/*w* to 0.25% *w*/*w* drug loading was achieved [6]. This limitation motivated researchers to change the manufacturing method, which eventually led to the development of filaments that were not simply wetted by the API solution but directly contained the given API within the filaments through hot-melt extrusion (HME). Although these early attempts reported higher drug loading percentages, the quality of the produced filament was not suitable for 3D printing. The authors declared that the use of other excipients as plasticizers could help to obtain considerably higher drug loading percentages than 10% *w*/*w* [7].

Since then, HME has become a widely used technology not only in the pharmaceutical industry but in other fields of industrial manufacturing. Presently, a variety of commercially available drugs are produced by this method, for example, the Lacrisert^®^ ophthalmic insert, Zoladex^®^ injectable implant, Implanon^®^ implant, NuvaRing^®^ vaginal ring, and Eucreas^®^ film-coated tablet. HME can be used for the manufacturing of granules, pellets, tablets, transdermal systems, transmucosal delivery systems, implants, solid lipid nanoparticles, or nanocrystals. It can be applied for solubility and bioavailability enhancement, taste masking, and co-extrusion. Targeted and shaped drug delivery systems, nanopharmaceutics, and filament manufacturing can be performed [8], and also, co-crystal formation can be achieved [9]. Using HME for pharmaceutical applications offers numerous advantages, as it has the potential to create new and novel drug formulations, and it has the ability to be connected to process analytical technology (PAT) [10]. In the case of solid dispersion manufacturing, the process does not require any use of solvent [11]. Some other advantages of using HME in pharmaceutical applications include shorter production time, higher process efficiency, and increased drug delivery efficiency in patients. Restricting factors include the high energy input, which is required for the shear forces during the manufacturing process [12]. Additionally, as HME also uses high temperature, it poses a risk of thermal degradation of the API [13].

Traditionally, HME-FDM technology utilized thermoplastic synthetic polymers, like polylactic acid (PLA), polyvinyl alcohol (PVA), polycaprolactone (PCL), acrylonitrile butadiene styrene (ABS), and high-impact polystyrene (HIPS) [14]. Pereira et al. listed the most routinely used polymers: PVA; polyvinylpyrrolidone (PVP); cellulose-derived polymers such as ethylcellulose (EC), hydroxypropylcellulose (HPC), hydroxypropyl methylcellulose (HPMC), and HPMC acetate succinate (HPMCAS); acrylates such as Eudragit^®^ E PO, RL, RS, and L; and other polymers like Soluplus^®^, Kollicoat^®^ IR, PCL, polyethylene oxide (PEO), or ethylene vinyl acetate (EVA) [15]. In the last few years, natural polymers have become more popular due to sustainable approaches. Porwal et al. converted biopolymers like chitosan, cellulose, or hemicellulose into printable polymers [16]. Even the possible use of nanocellulose was investigated due to its biocompatibility, good printability, and biomanufacturing potential [17].

Quality by Design (QbD) is an approach which emphasizes that the quality should be designed in a product prior to manufacturing, based on the observation that most quality problems originate from the way in which the product was designed. The U.S. Food and Drug Administration (FDA) encourages risk-based approaches and the adoption of QbD principles in drug product development, manufacturing, and regulation. In this article, the final quality attributes of the HME products were determined to be as follows: extrudate density, length/thickness/diameter, polymorphic form and transition, content uniformity, and throughput. Meanwhile, the input material attributes which are important are particle size and distribution, fines/oversize, particle shape, melting point, density, solid form/polymorph, and moisture content. The process parameters are the following: screw design, screw speed, screw opening diameter, solid and liquid feed rates, feeder type/design, feed rate, number of zones, zone temperatures, and chilling rate (Table 1) [18].

In the case of 3D-printed pharmaceuticals, the main principles of QbD will be the same: the identification and control of the critical quality attributes (CQAs), critical material attributes (CMAs), and critical process parameters (CPPs) to ensure consistent product performance and safety and provide batch-to-batch consistency. Even though the process should start by first determining the quality target product profile (QTPP), this might be challenging due to the lack of CQAs, CMAs, and CPPs. Prior to the publication of this article, the authors did not find any review articles which would specifically determine the key issues for the HME-FDM process [19].

Later in this review article we aim not only to summarize the importance of the properties listed in Table 1, based on publications in which HME-FDM was used for manufacturing drug delivery systems, but also to draft a guideline in harmonization with the QbD approach to facilitate easier drug development, manufacturing, and registration.

## 2. Materials and Methods

### 2.1. Review Process

This systematic review was conducted based on the guidelines outlined by the Preferred Reporting Items for Systematic Reviews and Meta-Analyses (PRISMA 2020, international guideline, PRISMA Group) [20,21]. Given the technical nature of the review, clinical risk of bias tools were adapted to assess methodological and reporting quality. The review process was performed independently by three authors who were involved in the writing (P.A.; Á. P.; and D. N.). The protocol for this systematic review was not registered.

### 2.2. Search Strategy

In this review article, a comprehensive electronic search was conducted through PubMed from 1 December 2025 until 29 January 2026. The database was used to identify studies investigating the application of Quality by Design principles in HME- and FDM-based drug dosage form development.

The search included combinations of the following keywords: “hot-melt extrusion” AND “drug delivery system”; “fused deposition modelling” AND “drug delivery system”; “hot-melt extrusion” AND “drug dosage form”; “fused deposition modelling” AND “drug dosage form”; “hot-melt extrusion” AND “ Quality by Design”; and “fused deposition modelling” AND “ Quality by Design”.

Automatically, all original research articles and review papers were selected from 2015 to 2025 which included any connected keyword in their abstract or title. All retrieved records were imported into Zotero (version 7.0.3). Before assessing eligibility, duplicates were removed with Zotero.

### 2.3. Eligibility

Screening began based on the titles and abstracts of records not directly related to pharmaceutical manufacturing or only mentioning but not focusing on HME or FDM. After the exclusion of such items, we aimed to access full text records for retrieval, and non-accessible publications were discarded. Disagreements on exclusion/inclusion among the three authors were resolved by consensus.

### 2.4. Study Assessment

All retrieved records were assessed for eligibility. We excluded publications where less than 3 process/material parameters were monitored or the Materials and Methodspart was incomplete, as important details were missing for either how the parameters were measured or the process could not be reproduced based on the description.

### 2.5. Data Extraction

One main aim in this study was to identify the HME-FDM-specific attributes which could be determined to achieve a higher-quality and reproducible drug delivery system. Data extraction was performed by using a standardized data collection form, extracting equipment, process, material parameters, and performed analytical tests in the case of both the HME and FDM sections of the selected studies. Based on the previously published articles, in Section 3.2 Hot-melt extrusion, HME equipment details and all published parameters, the used polymer type, and API were collected; and Section 3.3. Fused-deposition modeling summarized the used material, machine, and process parameters of FDM. The reported pre-analytical tests and intermediate plus final product characterization tests of HME manufacturing were also added in the review. The data for all three were summarized in three separate tables at the end of each section.

### 2.6. PRISMA Flow

The study selection process described in Section 2.2, Section 2.3 and Section 2.4 is summarized in a flow diagram following PRISMA guidelines in Section 3.1. A total of 1679 records were identified through database searching. After screening and eligibility assessment, 26 studies were included in the final analysis (Supplementary Materials).

### 2.7. Assessment of Reporting Quality

All included papers were assessed for quality of process/material parameter reporting. Due to the descriptive and highly heterogeneous nature of the reported parameters across the included studies, a quantitative meta-analysis was not feasible. As the most influential parameters are not standardized among publications, publication bias and certainty of evidence could not be evaluated. Therefore, a narrative synthesis was performed, grouping the data into HME processing, FDM manufacturing, and analytical characterization. Because standard risk-of-bias tools were not suitable for the aim of the review, we developed the criteria ourselves.

Namely, the three writing authors independently assessed the reporting quality of the selected papers. The main aim of the assessment was to highlight the current reporting frequencies of different parameters among researchers.

The reporting of the following parameters was investigated separately: HME equipment type, die diameter, HME screw configuration, screw diameter, information on the feeder (type or speed), HME process temperature, HME screw speed, torque values during HME process, FDM machine type, nozzle diameter, printing bed temperature, infill, infill pattern, layer height, and printing speed. Parameters which were mentioned only in 3 or fewer publications were not graphically demonstrated, but nevertheless they are mentioned in the respective subchapters of the review.

Graphical results of the assessment of reporting quality can be found in Section 3.5. Based on the facts reported, publications were categorized either as “Yes” or “No”.

## 3. Results and Discussion

### 3.1. Study Selection

**Figure 1:**
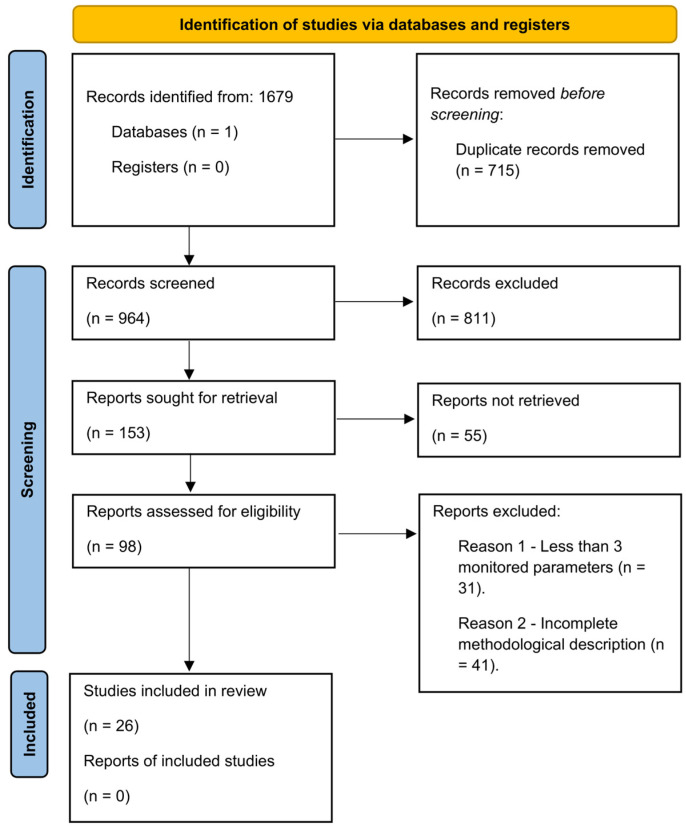
Preferred Reporting Items for Systematic Reviews and Meta-Analyses (PRISMA) flow diagram demonstrating the literature search strategy.

We identified 1679 records from our literature search on the databases, and after filtering duplicates, 964 unique original published articles were identified. Their eligibility was checked based on analysis of titles and abstracts, and a list of 153 articles was created from which we could retrieve 153 reports. Full texts of these shortlisted manuscripts were then examined, and based on the previously described exclusion criteria, a final selection of 26 eligible studies was analyzed in our review after application of inclusion and exclusion criteria. The literature identification strategy is demonstrated by means of a PRISMA flow diagram in Figure 1.

### 3.2. Hot-Melt Extrusion

#### 3.2.1. HME Method

**Figure 2:**
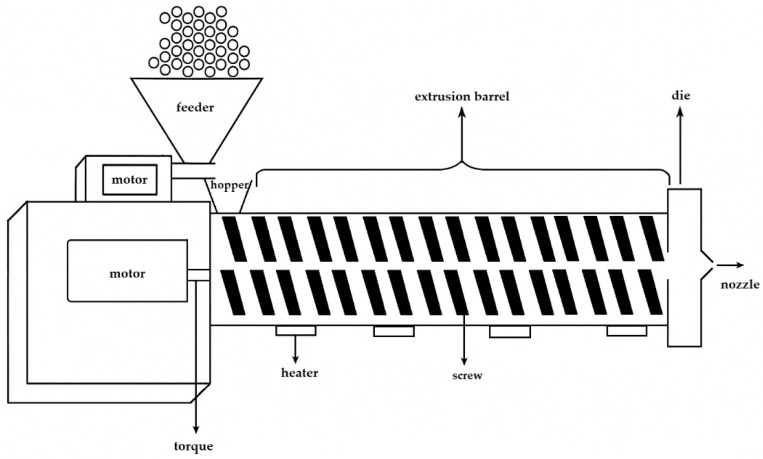
The schematic figure of a hot-melt extruder and the main parts. The figure was generated by the authors with Paint.NET 5.1.10.

For filament manufacturing, a feeder, an extruder, a conveyor belt, and a winding machine are needed. The feeder provides continuous solid material flow. The main part is the so-called extruder, which consists of a motor, a hopper, an extrusion barrel, the screw(s), and a die (Figure 2). During the continuous process, the solid sample enters through the hopper and reaches the area of rotating and heated screw(s) inside the barrel. The rotation speed can be controlled, and, based on the type of manufacturer, different heating zones can be applied. The heaters provide the thermal energy for melting, and the screw(s) provide shear stress and intensive mixing of the material. At the end of the barrel a die is placed with a given diameter. On the conveyor belt the filament solidifies, which can be supported by active cooling, and the diameter of the filament can be adjusted by the speed of the conveyor belt. Finally, the filament is wound by the winding machine and is ready to use for the FDM printer [10].

#### 3.2.2. Types and Screw Parameters

**Figure 3:**
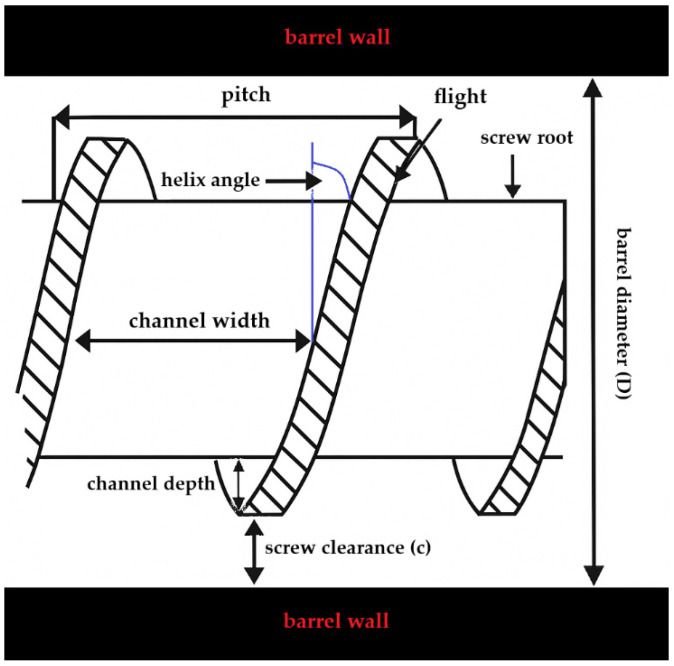
The schematic figure of the screw and its most important parameters. The main parts are the barrel wall, the screw root, and the flight. Based on the equipment the following parameters can be altered: barrel diameter (D), screw clearance (c), channel depth, channel width, pitch distance, and helix angle (labeled with blue color). The figure was generated by the authors with Paint.NET 5.1.10.

Hot-melt extruders can be divided into subgroups based on the position of the screws. Extruders are designed as single-screw, twin-screw, or multi-screw extruders, and must meet regulatory standards for industry-sized manufacturing [22]. The twin-screw extruder can be further divided into those with counter-rotating or co-rotating screws. The dimensions of the screws are normally defined in a length-to-diameter (L/D) ratio. The screws are mostly made from stainless steel, and the main parts are the screw root, flight, and barrel wall. Based on the equipment, the following parameters can be altered: barrel diameter (D), screw clearance (c), channel depth, channel width, pitch distance, and helix angle, as can be seen in Figure 3 [10].

#### 3.2.3. HME CMAs and CPPs

According to our research, different authors reported various, often contradictory HME CMAs and CPPs. In one article (which can be found in Table 1), the CPPs of the HME equipment were identified as screw design, screw opening diameter, feeder type/design, and number of zones [18]. In another article, no equipment data were highlighted [22]. Bandari et al. determined in an Ishikawa diagram that the most important attributes based on the HME equipment details are screw elements, screw configuration, die diameter, die shape, and barrel volume [23].

In the existing articles the CPPs were defined differently. For example, the CPPs of the HME process parameters were determined in Table 1 as the following: screw speed, zone temperatures, feed rates, and chilling rate [18]. In another paper the key parameters of the extrusion zone which can be modified were the following: the temperature of the zones, the rotation speed of the screw(s), the feed rate, and the speed of the conveyor belt while the filament is cooling down [8,10]. Patil et al. also determined the critical process parameters (CPPs) of HME-FDM, but they determined fewer parameters: process temperature, extruder pressure and torque, feed rate, and screw speed were selected as influencing the data [22]. Bandari et al. described the same, but instead of the extruder pressure the die pressure was mentioned [23].

Regarding the CMAs, the particle size and distribution, fines/oversize, particle shape, melting point, density, polymorphic form, and moisture content were determined as important parameters [18]. In another article, the API and polymer miscibility, the drug load, the viscosity, and thermal behavior, like thermal expansion, glass transition, and melt and degradation temperature, were also highlighted [23].

The used polymer can also be an influencing factor for the outcome of the HME process. Regarding the selection of the polymer, we must consider some properties of the polymers, such as if they are suitable for pharmaceutical applications, whether the API is stable at the melting point of the polymers, and whether it is necessary to use any plasticizers, as they can affect the biocompatibility [15].

Finding convenient raw materials can also play a crucial role, as the filament stiffness and brittleness could be adjusted significantly by changing the type of the release modifiers. Moreover, in vitro drug release studies have revealed that the drug release could simply be controlled over 24 h by only changing the type of the release modifiers [24].

#### 3.2.4. Literature Overview About Manufacturing and the Used Process Parameters

In this literature overview section, we will further explain the above-mentioned conceptualization about the CMAs and the CPPs previously stated in different articles. The authors in the selected articles searched for the described HME equipment, and the process details, the used polymers, and the APIs are summarized in Table 2. In subsequent sections, the authors provide a descriptive analysis of the included 26 studies, as the critical comparison revealed some consistent trends in the applied equipment details and process parameters which could determine the CMAs and CPPs of the HME.

First, the described HME equipment details were compared. Of the 26 articles, 3 did not mention the used equipment, 1 stated it was an experimental one, and 2 used a single-screw extruder, which means that 20 researchers used twin-screw extruder. Regarding the type of the twin-screw extruder, 2 were counter-rotating, and 11 were co-rotating; in 7 cases the rotation was not mentioned.

The die diameter was stated in 17 articles, and the range was between 1.5 mm and 2.7 mm. The number of heating zones was mentioned in five articles, and in the chosen articles the equipment contained eight heating zones.

The screw configuration was mentioned in nine articles; three used three mixing zones, and one used a kneading element. Data on three detailed and five standard screw configurations were provided. The screw diameter was given in seven articles, and the values ranged between 10 and 12 mm; the screw L/D ratio was given in six cases, and the values were 20 or 40.

The feeder was mentioned only in four articles; in two it was manual, in one it was only mentioned, and in one the exact feeder rate was given as 50–100 g/h.

The available data in the reviewed publications about the HME equipment details and the used parameters varied significantly. Most studies utilized a twin-screw co-rotating extruder, and if it was mentioned, a standard screw design with three mixing zones was used. Some articles showed the screw set-up with illustrative diagram, while others left it unclear whether the device had one or two screws. Even with the fact that more recent works show a shift in the amount of data provided, there is a critical gap in the available information, which limits comparability and reproducibility across studies. Based on the articles, we believe that integrating the QbD approach and the CPPs would be crucial to determining the quality of the product.

The HME parameters were reported in the examined 26 articles as follows: Temperature was mentioned in all articles (m = 26) and screw speed was mentioned in 24 cases. The torque value was mentioned in 5 articles, the residence time in 1, and the feed rate in 2. In a single article it was mentioned that a cooling fan was required and that for the cooling a conveyor belt was used, and the pressure, drive, and SME were also given.

All studies mentioned the temperature, but it was not clarified if it was the same in all zones. Earlier papers treated temperature as the primary degradation risk [1,26], while Hoffmann et al. demonstrated that residence time and shear behavior were equally decisive [37].

The screw speed was reported as an important CPP before by separate research groups, but in two cases the data were not reported.

A research group recorded the torque values (N·cm) and plotted them against the drug content percentage, and they changed the extrusion temperature between 100 and 120 °C by changing it 4 °C in each step. The study revealed that the torque value was inversely proportional to both the extruding temperature and the drug content. Specifically, as the extruding temperature or API content increased, the torque value decreased. This effect was particularly pronounced at drug content levels of 16–18%. This phenomenon can be attributed to the plasticizing effect of ibuprofen on the EC used in the research. At an extruding temperature of 120 °C, the relationship between torque and drug content was nearly linear [29].

One more important aspect is that the FDM printer is only able to manage filaments with exact diameters. For this, Holländer et al. published the first article which adjusted the diameter of the extruded filament by changing the speed of the conveyor belt. If the speed was increased, the filament diameter would become smaller [25]. Another research group used this technique as a fine-tuning method [30].

Additionally, the authors emphasized the importance of maintaining a controlled environment (e.g., ambient temperature and humidity) to further improve the outcomes of the printing process [30].

In another article, the authors suggested the development of a software to calculate CPPs, as it aligns with the QbD approach [30].

Some relevant material attributes were also mentioned in the examined articles. Regarding the used polymer and the API, the authors had the option to use a variety of materials which will affect, from formulation to formulation, the appropriate HME parameters. In an article, ten different polymeric components were examined, and the authors stated that even though all products were suitable for 3D printing, the strict quality standards required for industrial feasibility must be considered for future use [1]. The used API amount can also be an influencing factor through the manufacturing of the filament. In an article where 5%, 10%, and 15% indomethacin were used for the manufacturing of an implantable medical device, higher drug loading resulted in poorer quality. As a result, it was stated that with the use of lower drug content, better quality and a higher dissolved API amount could be achieved [25]. In another article, a maximum of 60% API could be used with PEO, and a higher amount of API required a higher extrusion temperature. Also, the type and amount of the applied surfactants required different HME parameters [35].

The use of thermo-sensitive APIs also played a crucial part, as in the case of enalapril maleate [37,38]. Two studies directly contradicted the earlier assumption that such drugs are unsuitable for HME [1,26]. Lower temperatures alone are insufficient; short residence time and optimized screw design enabled successful processing [37,38].

#### 3.2.5. Other Relevant Information

In this section the authors of this review would like to highlight some other relevant article which described important information about CMAs or CPPs.

**Figure 4:**
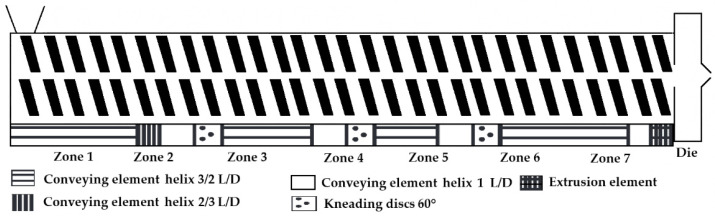
A detailed screw configuration example. The figure was generated by the authors with Paint.NET 5.1.10.

As the screw configuration can also play a crucial role, a standard configuration was displayed in a figure by the authors for better understanding. Even though no significant statistical result can be determined, in all cases, when it was mentioned, the researchers used eight heating zones and a standard or detailed screw configuration with three mixing or kneading zones, which is also visualized in a figure (Figure 4) [49].

The same research group showed that regarding the attributes of screw speed, powder feed rate (PFR), and barrel filling degree or specific feed loads (SFL), only the last one could be identified as key parameter for diameter uniformity independently of the set PFR–screw speed combination. An SFL of >0.04 led to filaments with acceptable diameter quality according to European Pharmacopoeia (Ph. Eur.) uniformity of mass standards. The higher the fill level, the lower the fluctuation of the mean diameter was [50]. Based on a previous article, the effect of “die swell” was determined, which means that the diameter of the filament will increase upon leaving the die due to the viscoelastic properties of the polymers, just like in case of tableting [22].

One of the most detailed descriptions of the HME could be found in the article of Patel et al., where specified screw configuration data with exact kneading element geometry and position, as well as offset angle, all barrel and die temperatures, feed rate, screw speed, and die diameter, were given. In this study, it was stated that the temperature in the feeder zone was set to 50 °C to avoid premature melting of materials, a failure which could block the powder flow from the feeder to the barrel [49].

In some articles, before the HME process, the homogenization of raw materials was performed, but only in rare cases was sieving used. A research group highlighted that even though the HME has mixing functionality, adequate pre-mixing or homogenization reduces the batch-to-batch variation [22]. As an example, a V-shell blender was used at 25 rpm for 20 min, and then the material was fed into the extruder [45]. In a study, the choice of polymer and degree of particle size reduction of the extrudate by milling were the two key influencing factors on the release profile, and it was determined that the smaller the particle size, the slower the dissolution time, a result that was largely independent of drug loading and robust to the hydrodynamics of the dissolution vessel [51]. In another article the mixtures were milled to five sieve cuts ranging from <45 μm to 355–500 μm; however this had no effect on the final product. The particle morphology was highly dependent on drug loading and HME operating temperature, as the placebo cases yielded higher-aspect-ratio particles in contrast to the lower-aspect-ratio particles of the API and polymer combinations. The HME temperature determined whether residual crystallinity would be present or absent in the extrudates, which impacted the surface roughness of the particles [52].

Regarding the manufacturing site conditions, the temperature and humidity throughout the extrusion process were monitored in another research article, and it was mentioned that after HME, the filaments were stored on a spool at room temperature in a drier at a relative humidity of 40% before being used for further analysis [13].

As a new approach, some research groups utilize vacuum compression modeling (VCM) to enable faster iterative development cycles, even though a significant knowledge gap exists in predictable and scalable transitioning [53]. This technique can be used by the researchers as a preliminary tool for optimizing formulations before proceeding with HME [54].

Based on the data available to us, the authors’ opinion is that despite the technological maturity of HME for pharmaceutical filament manufacturing, the reporting gaps remain a major barrier to process standardization, scaleup, and regulatory translation.

### 3.3. Fused-Deposition Modeling

#### 3.3.1. FDM Method

An extrusion-based process known as fused deposition modeling (FDM) 3D printing uses filament extruded through a heated nozzle which is then spread out on the build plate in successive layers, where they solidify. This 3D printing technique allows the formulation of various dose forms [55]. The advantages and limitations can be found at the beginning of the Section 1.

#### 3.3.2. Parameters

The parameters of 3D printing can be divided into two subgroups: one is the process, or usually called printing, parameters, and the other one is the machine parameters, which can be changed.

In the case of FDM printing, the process or printing parameters can be changed prior to the 3D printing in the software. As in the case of HME, the chosen parameters are varying, and an exact conclusion cannot be made by the authors of this review; our aim is only to highlight some of the concepts.

In a research article, the most often changed parameters were determined as infill density and infill pattern, extrusion or printing or nozzle temperature, layer thickness, and raster angle, and all the reasons for these changes were settled by the article [56]. In another research article, the nozzle and platform temperature, the printing speed, and the layer thickness were highlighted [57].

The infill density could be varied from 0–100%, where 0% means hollow, and 100% means solid parts in the printed object, and the load-bearing capacity of the printed object should increase when increasing the amount of the material inside the printed object [58]. Some research groups examined the effect of the infill percentage on the dissolution profile. A study showed that 20% infill demonstrated the fastest and most complete release, whereas 80% infill resulted in a more controlled release, and the performance and dynamics of the drug release can be changed without changing any of the formulation parameters (drug load, polymer, and composition) [59]. Not only the infill percentage, but the used infill pattern will affect the mechanical properties of the part, but the direct influence could not be determined yet [56].

Ilyes and co-workers analyzed the effect of shell and layer thickness on the accuracy and the precision of the manufactured tablets. In the study, carvedilol-based filaments were used, and it was found that using a higher shell thickness (0.9 mm) and lower layer height (0.1 mm) led to better accuracy in the diameter and height of the tablets [60].

In a research article, certain FDM process parameters like raster orientation/raster angle and raster width were analyzed much more than some other process variables like interior infill pattern, infill density, temperature of extrusion, number of contours, etc. Based on the authors’ opinion, the majority of optimization is only carried out as a single output variable, or there is optimization of multiple-output variables, but each is optimized one at a time and not simultaneously. Thus, further research needs to be performed for optimization by not only statistical-based techniques, but utilizing image processing, machine learning, and deep learning processes [61]. Consequently, careful optimization of the software would be critical to ensure the desired quality and reproducibility of the printed dosage forms [62].

In the case of FDM printing, some of the machine parameters of the equipment can be changed prior to the printing; meanwhile, for the process parameters, nothing can be done, as the 3D printing takes place automatically.

The build orientation could be guided by the step where the part is oriented toward the X, Y, and Z axes on the build platform, but this requires adequate platform leveling to be performed prior [63].

The nozzle diameter depends on the machine type, but usually the FDM printers are provided with four nozzle diameters: 0.2 mm, 0.4 mm, 0.6 mm, and 0.8 mm. In the article of Macedo et al. the effect of the nozzle size was investigated. As a result, it was said that with the use of a smaller-diameter nozzle, higher resolution could be achieved, and a lower mass average was obtained due to better control over the material flow. Also, changes in the porosity and drug release rate were observed using different nozzles; however, no clear trend could be detected [64].

The filament diameter is also an important parameter, and as mentioned in the HME section, the exact or necessary filament diameter can be adjusted with the speed of the conveyor belt.

The feedability and printability raise one of the big questions for the researchers, as having the ability to manufacture a filament does not necessarily mean we can print it [65]. The mechanical flexibility was also reported in the work of Repka and Zhang, and a specified method for HME-FDM filaments was set up for the evaluation of brittleness and stiffness mechanical test results [31,66].

Rheological characterization is also important, as viscosity predicts the printability, controls the deposition process, and ensures layer adhesion [67]. Consequently, the melt flow property of a material, which is temperature-dependent, is a critical process parameter that can significantly impact the printability and quality of the printed object [30].

These findings indicate that feedability and printability are multifactorial properties, arising from the interplay between rheological and mechanical characteristics, which may not always be directly correlated. In the authors’ opinion, for the feedability, the mechanical properties may play a more important role, but for the printability the rheology may be more crucial.

In another article, the environmental factors such as room temperature and humidity were highlighted as an influencing factor on the outcome of FDM printing [68].

#### 3.3.3. FDM CMAs and CPPs

According to our research, different authors reported various, often contradictory FDM CMAs and CPPs.

In an article, the quality and reproducibility effects of the FDM process were determined by the material parameters (rheology and flexibility) and the machine parameters, such as the chosen nozzle diameter, the manufactured filament diameter, the motor step size, and adequate platform leveling. Meanwhile, the process parameters were determined to be the nozzle and platform temperature, printing speed, and layer thickness. All these parameters could be set in the software of the FDM printer. According to the authors, the feedability is affected by the mechanical flexibility and filament diameter. The weight of the product is impacted by melt rheology, platform leveling, nozzle temperature, and printing speed. The dimensional accuracy could be influenced by the motor step size, the platform temperature, the printing speed, and the layer thickness. The road width, which is inversely proportional to dimensional accuracy, is influenced by the following parameters: melt rheology, the nozzle diameter, and the nozzle temperature [57].

In another research article, the CMAs were added as feedstock attributes, and the highlighted parameters were the surface morphology, the feed stock mechanical properties, the melt viscosity, the filament diameter, and the swelling/shrinking of the filament. Regarding the CPPs, the machine parameters were the nozzle size, the printing head location, and the number of printing heads. The process parameters were identified as printing speed, nozzle, and bed temperature of the 3D printer, and the parameters of infill density, dimensions, shape, infill pattern, and layer height were adjustable via the software of the printer [23].

In a review, the CMAs were identified as filament properties (e.g., mechanical, thermal), filament diameter, and API (e.g., filament stability, excipients compatibility), and the CPPs as nozzle and bed temperature and printing speed [69].

#### 3.3.4. Literature Overview About FDM Parameters

In this section, the authors summarize the reported material, machine, and process parameters in previously mentioned articles regarding the FDM process, which can be found in Table 3.

First, from the selected 26 articles, in 1 article, even though the article title and the abstract contained information about 3D printing, the actual 3D printing was not performed. However, the article contained valuable information on the QbD approach, and thus the authors did not eliminate this study. Because of this, in this section only 25 articles could be evaluated.

Regarding the material parameters, the authors faced an issue of reporting about the material parameters, as in the articles only the amount and type of the used materials could be found. Based on the CMAs the melt rheology and mechanical flexibility could be mentioned, which can be measured analytically, and the authors of this review added these tests in another section. Unfortunately, no other relevant material parameters were shared in the eligible articles.

From the machine parameters, the FDM printer type was reported in 24 articles, and in 2 articles nothing was mentioned. In 18 articles, the nozzle diameter was reported as 0.4 mm, except for one case where 0.6 mm was chosen by the researchers. The build plate material was mentioned in three articles, and in one article an unheated build plate was chosen.

In the FDM reporting, the process parameters gave us the most data. The printing temperature was mentioned in all cases, and the bed temperature was mentioned in 19 articles. In a publication, 13 different polymers were manufactured, and 8 were successfully processed into tablets by 3D printing. The authors noted that an increased temperature was needed to effectively print the filaments by the FDM method, as non-traditional filaments were used and consequently the shear stress was low. As a result, a good correlation could be seen between the tablet size and the mass of the tablet, which indicates the easy adjustment of dose and that the method is suitable for personalized drug delivery [30].

The infill was reported in 24 articles, the infill pattern in 3 articles, the layer height in 23 articles, and the layer or path or line width in 4 cases. The printing speed was mentioned in 19 articles, and the travel speed in 8 articles. Other reported parameters were the following: number of shells in three articles, and shell thickness and outside shell thickness in two articles. These parameters were reported in one article: path width, minimal layer time for cooling, outlines printed on each layer, floor and roof thickness, fan speed, shell thickness, and wall thickness.

In an article, it was noted that FDM 3D printer heads are difficult to clean and that the feedability of medicinal filaments was low, which could be solved with special FDM printers [36]. Even though the 3-point bend (3PB) test showed that some of the filaments were too soft or brittle, one research group performed the feedability test, and appropriate FDM printing could be achieved [26].

A descriptive analysis of the included studies revealed several consistent trends in the applied process parameters and equipment. Most authors found it important to clarify the exact nozzle size used, but mostly no other machine information was shared. All authors found it important to report the printing temperature, which is a material-dependent factor. Most studies utilized the bed temperature, the infill percentage, the layer height, and printing speed. In most articles, many other parameters were shared by the authors, but the reporting frequency for each parameter was heterogenous. However, a critical gap in the literature is the lack of homogenous parameter reporting, which limits comparability and reproducibility across studies.

### 3.4. Analytical Testing

#### 3.4.1. The Methods

As was mentioned above, in order to achieve proper quality, the input material characteristics should be examined, such as the particle size and distribution by laser diffraction or dynamic light scattering (DLS), and fines/oversize and particle shape by scanning electron microscopy (SEM), optical microscopy, or dynamic image analysis. Automated static image analysis could also be used for the characterization of particle size distribution and morphology, enabling quantitative evaluation of particle shape parameters [70]. The melting point and solid form/polymorphism could be examined by DSC and/or thermogravimetric analysis (TGA) [65]. The density can be measured by pycnometer, and moisture content by moisture analyzer, Karl Fischer titration, TGA, or the moisture sensor of a near-infrared spectroscope (NIR) [71]. Whether these methods are performed or not, some articles mentioned various pre-manufacturing steps, such as proper mixture of the powders or removal of residual moisture, which can affect the feeding capacity of the HME [24,36].

After production, the manufactured filaments must undergo multiple investigations. The characterization possibility of HME products is not a new approach and can be divided into five subgroups:

1. Physico-chemical characterization by PXRD, Fourier transform infrared spectroscopy (FTIR), NIR, or nuclear magnetic resonance spectroscopy (NMR),

2. Thermal characterization by DSC or TGA,

3. Microscopic characterization by SEM, micro-computed tomography (micro-CT or µCT), X-ray computed tomography (XRCT), hot-stage microscopy (HSM), or polarized light microscopy (PLM),

4. In vitro dissolution testing [72].

The above mentioned physicochemical, thermal, and microscopic characterization methods are extensively discussed in earlier reviews [73,74,75].

5. Other HME-FDM-specific tests will be described in the next paragraphs which are necessary for proper characterization but were not mentioned in the previous articles: during or after HME, filament diameter determination should be performed; after that manufacturing come the mechanical characterization and FDM feedability and printability tests, finishing with the contact angle measurement of the printed samples. The rheology measurements could be performed with the polymer, the manufactured filament, and/or with the manufactured samples, if it is applicable.

Filament diameter measurement could be performed offline by digital or manual caliper. We also need to mention the in-line diameter and ovality determination by a laser-based module. Ponsar et al. used a laser module, and the diameter was recorded in three different directions with a 1 Hz sampling rate. The ovality of filaments was calculated as the difference between the maximum and minimum determined diameter per second [50].

For the mechanical characterization, the researchers mostly use a texture analyzer with different methods or parameters. For these experiments Patil et al. wrote that the 3PB test and Repka–Zhang test must be performed in the case of HME, where Hooke’s law can be assumed to describe the stress–strain relationship [22]. A different research group compared the 3PB, resistance, and stiffness tests [66]. The 3PB test is a classic experiment in mechanics and is used to measure the Young’s modulus of a material in the shape of a strip, bar, or stick, for example. The material is fixed on two supports and targeted in the center with a load. In the work of Repka and Zhang, a specified method for HME-FDM filaments was set up for the evaluation of brittleness and stiffness, as the printability of the filaments could be predicted from the results [31]. In a research article, the resistance test was also performed by a texture analyzer. The filament was cut into a certain length, then attached vertically to a cylinder rig and on the bottom to the hole of the sample holder. The maximum force and fracture distance were recorded [76]. The stiffness test was performed by Zhang et al., and the texture analyzer was equipped with a three-point-bending rig with thin blades, and the filament was placed directly on the platform, which in this test was a solid, flat metal surface. The blade penetrated the sample with a 35% change in shape/deformation (0.6 mm), and breaking stress and force data were collected [31]. In another article, the tensile stress of filaments was determined by pressing the filament into film with a high-pressure flat heat press machine, and this film was pressed into a dumbbell shape using a standard cutter. The initial width and thickness were determined. These dumbbell-shaped samples were also vertically stretched by a self-made device using constant force. The ultimate load was recorded, and the tensile stress at the breaking point was calculated [29].

A research group performed a special test with a texture analyzer which can determine the forces to which the filament is exposed in the FDM printer head. For this measurement a standard texture analyzer was used and equipped with an in-house rig and a 5 kg load cell. Filaments were compressed axially with a speed of 3.15 mm/s, corresponding to the roller movement speed of an FDM printer. The compression distance was set to 15 mm with a trigger force of 0.05 N. As data were collected during both compression and release, the areas under the curves could be determined and plotted to make comparisons of the stress on the filament vs. the distance travelled by the probe. The non-feedable filaments all shared a characteristic brittle fracture pattern, with sudden discontinuation of force on the filament after reaching a peak fracture force [76].

FDM feedability and printability tests could also be performed. Xu et al. used a cylinder-shaped tablet as a model. The manufactured filaments were printed with the same printing parameters. If the filament could be fed and printed more than three times, the filament was labeled ‘Yes’ for printability [66].

The contact angle measurements provide insights into the surface properties of the materials and can be easily measured by a goniometer. The wettability has an important role in the aspect of API liberation: a hydrophobic polymer decreases the wettability, so it has the potential to prolong drug release. Also, the wettability can affect the rejection of implantable systems [47,62,77].

For the rheological characterization, both capillary and oscillatory rheometers could be used [67]. Saba et al. performed characterization according to different standards such as ASTM D358 or ISO 14129 [78]. Rheological characterization of the API containing samples plays an important role, as it has the potential to indicate incomplete thermal homogenization within the nozzle of the 3D printer. Non-classical methods, such as performance X-ray particle tracking velocimetry (XPTV), could be used to reveal significant deviations from classical Newtonian flow, highlighting complex heterogeneous and non-isothermal behavior within the melt. It could be used as an in-line characterization if tungsten powder as tracer particles were embedded into polymer filaments [79]. Rheology measurements could be used to determine the viscosity. In a study, HPC-based formulations were manufactured, and as a result it was found that the viscosity of the printable blends could be as low as 10–1000 Pa·s at 100 rad/s angular frequency. The addition of 30–60% drug or disintegrant tended to lead to greater viscosity values. This rheological data could be useful for pre-formulation [80].

#### 3.4.2. QbD and Analytical Testing

The authors of this review suggest dividing the analytical testing into three subgroups regarding when it could be used: pre-analytical, intermediate, and final product analytical testing. Some methods could be used in all three steps, such as DSC, TG, or PXRD, but some of them are specific, such as mechanical testing, rheology, and contact angle testing.

Regarding QbD and pre-analytical testing, in a few articles, the CMAs for the HME process were mentioned, about which we have provided information, as detailed in paragraph 3 of Section 3.2.3. The authors of this review did not find detailed descriptions about which analytical methods should be performed and what consequences they can have on the quality of the final product. Regarding HME-FDM, it should be kept in mind that we also need to examine the quality attributes of the intermediate product obtained from HME, which the authors would like to call intermediate product analytical testing.

Yu et al. noted that the extrudate density, length/thickness/diameter, polymorphic form and transition, content uniformity, and throughput should be examined [18]. In the previously mentioned work of Xu et al., the lack of quantitative methods for the evaluation of HME products was criticized [66].

Final product analytical testing is the most mentioned and performed step, and most of the methods mentioned in Section 3.4.1. could be used as a final product analytical testing methods.

#### 3.4.3. Literature Overview About Analytical Testing

In this section, the authors summarize the reported pre-analytical tests, intermediate tests, and final product characterization tests in the examined articles, which can be found in Table 4. The pharmacopeial dosage-form tests e.g., dissolution testing and uniform mass testing, were not included in this section, as in the articles, different drug delivery systems were manufactured, and the unique tests could distort the results.

First, the described pre-analytical tests were compared. Of the 26 articles, 10 used PXRD for the raw material determination, while FTIR was performed in 2 articles.

In 19 articles DSC was used, and in 10 articles TGA or derivative TGA was performed. In an article, TGA was mentioned as a zero step for their experiments; the raw polymers were tested before the HME process [34]. Based on an article, it could be stated that HME should be performed at temperatures 20–40 °C above the melting point of the polymer. Furthermore, the API melting point must be considered. Nevertheless, some polymers, such as PCL, can pose challenges in achieving a uniform mass, which is a critical process parameter [18].

Rheology was used in two articles; in one article a shear rheometer was used, and in another the melt viscosity was determined by a rotary rheometer. In one case the known viscosity of EC was added, but we excluded it because a measurement was not performed by the authors.

In two articles, the particle size and/or distribution was mentioned: in one article the particle size of the powder mixture was determined, and in another the raw material particle size and particle size distribution were mentioned.

PLM, SEM, and VCM were performed in just one article. In one research article, some materials were kept in an oven at 40 °C for 24 h prior to use to decrease the moisture content. In 3 articles of the 26, no pre-analytical testing was mentioned.

Regarding the pre-analytical testing of HME-FDM manufactured products listed in this review, we found that the most frequently used, but not mandatory, tests involve raw materials, such as PXRD, DSC, and TGA testing. Only a limited number of articles were available where other methods were described, and their utilization was not heterogenous even though the CMAs were described in different articles, but also different attributes were selected by the authors.

Regarding intermediate product testing, PXRD was performed in 15 articles, FTIR in 2 articles, and scanning Raman and in-line NIR in 1 article. DSC analysis was used in 19 articles, and TGA or DTGA were used in 9 articles.

Versatile microscopic imaging techniques were sometimes chosen, and in total nine articles mentioned any methodology. SEM was used in six articles, stereoscopic microscope and PLM in two articles, and optical microscopy, elemental analysis, Keyence digital microscope, and EDX in one article.

Rheology was investigated in three articles, and DVSA and in-line diameter determination were used in one article.

Mechanical testing was mentioned in a total 16 articles, but the performed methods were versatile: 3PB was used in 10 articles, Repka–Zhang in 4, stiffness testing in 2, and in 3 articles differently named mechanical tests were performed. In some articles, different mechanical tests were performed.

In one article, the conclusion was made that polymers with higher molecular weights led to high stiffness. On the other hand, polymers with lower molecular weights form filaments that break easily. The results of texture analysis can be used to determine whether the filament is printable; however, the quality of printing cannot be distinguished based on this analysis [40].

Particle size determination within a manufactured filament can be challenging. As a result, a research group decided to perform analysis by scanning Raman microscopy and found in the cross-cut scans that the alignment of the particles with the three-dimensional flow inside of the extruder barrel was depicted by two vortices around the screws. Higher-particulate drug load results in higher viscosities, which cause elevated process parameter values for SME and nozzle pressure. This higher energy input fosters the comminution of particles, resulting in higher specific interfacial areas in the composites and thus smaller mean distances between particles. The distance for crack propagation is reduced as well, which shifts the mechanical properties of the filaments from elastic ductile to rigid brittle behavior with increased drug load [44].

Final product analytical testing was not mentioned in 4 articles of the 26 selected articles. PXRD was used in 14 articles, FTIR in 2 articles, and NIR in 1 article. DSC was performed in 17 articles; meanwhile TGA or DTGA was performed in 8 articles.

Versatile microscopic imaging techniques were chosen as a final testing method as well, but in total only 16 articles mentioned any methodology. SEM was used in 12 articles, stereoscopic and optical microscopy and PLM in 2 articles, and elemental analysis, a Keyence digital microscope, and EDX in 1 article.

Contact angle measurement was mentioned in just one article.

The final product analytical testing in the above listed publications can be sub-grouped regarding the type of test. Of the physico-chemical tests, PXRD was used in almost all the cases, and in some publications FTIR was also used. Thermal analysis was performed usually both by DSC and TGA. Some type of microscopic analysis was also performed by SEM, EDX, or PLM.

### 3.5. Assessment of Reporting Quality

**Figure 5:**
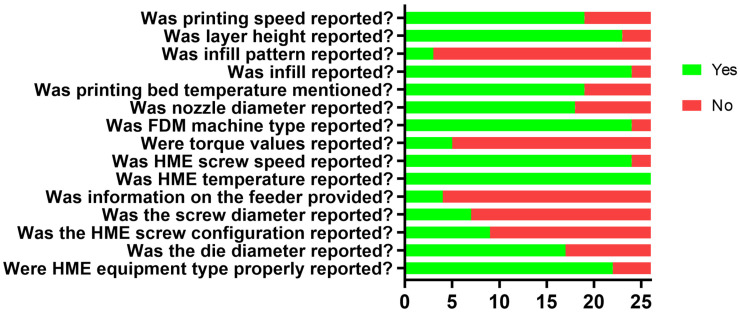
Assessment of reporting quality analysis results.

An assessment was carried out to visualize the most frequently reported HME and FDM process parameters in the selected studies, depicted as a stacked bar-chart in Figure 5. The results further emphasize that there is no “standard list” of reported parameters. While temperature can be considered as a triviality alongside API and polymer type, even HME equipment type is not always mentioned. Such crucial information as what type of feeder or what speed was utilized or what nozzle diameter was used are rarely mentioned. These could have been easily reported, as they are readily available parameters compared to torque, which can only be monitored with specialized sensors which might not even be possible to implement with the chosen manufacturing equipment. A more robust, detailed publishing practice must be implemented by researchers to provide information on these seemingly trivial parameters which have tremendous effect on the manufacturing process and product quality. Neglecting any vital parameter prevents not only the development of well-established QbD models but the improvement of this field, as the missing information could possibly solve certain issues faced by researchers.

### 3.6. Pharmacopeial Dosage-Form Tests

Even though pharmacopeial dosage-form tests were not discussed in this review, it is important to mention that the results of an API dissolution test, as one of the important feedback parameters, can reveal correlations with appropriate quality.

In an article, the researchers were interested in how the HME can affect the results of the dissolution profile, so the aim was to manufacture a tablet traditionally by mixing and compressing (“physical mixture”); meanwhile, with the same formulation, a hot-melt extruded filament was manufactured, and some part of this filament was 3D printed (“printed tablets”), while the rest was re-granulated and compressed by a tableting machine (“HME tablets”). As a result, it was stated that the 3D printing method decreased the variability of the dissolution profile. The reason for this is mainly the increased hardness of the printed tablets compared to the other two methods used [42].

A research group intended to make two different filaments. The core filament consisted of 55% PVA, 13% mannitol, 7% sodium chloride, 5% PVP K30, and 20% diltiazem; mannitol was used as a plasticizer and osmotic agent, and sodium chloride was used to enhance the osmotic properties. The shell filament was made from cellulose acetate (CA) and 25% triethyl citrate as a plasticizer. From these two filaments, three different digital designs were made to determine the effect on the 3D printing process. The first formulation had a no-top, the second formulation had an imported hole and one linear cavity, and the third formulation had two linear cavities. The first formulation showed an immediate release profile with quadratic kinetics, and the other two showed delayed releases. However, the API dissolution started faster with the third formulation, which consisted of two linear cavities [32].

Using HME-FDM, Zhao et al. developed a special floating and sustained-release tablet containing venlafaxine hydrochloride, which had a zero-order drug release profile for 24 h. The design featured an insoluble PLA shell and a drug-loaded core. The PLA shell contained a hollow-structured air chamber at the top to maintain the floating property and a drug release window at the bottom with an adjustable diameter. By varying the amounts of materials, 3D printing parameters, and the STL file design, the drug release could be adjusted as needed [39].

Another study investigated the relationship between tablet surface area and drug release rate, highlighting how surface geometry could be used to modulate dissolution profiles. By correlating tablet design parameters with in vitro release, the work demonstrated that 3D printing allows precise control over drug release kinetics [46]. In a literature review, 56 products were reported; 43 had sustained or delayed release, 4 were developed for both immediate and sustained release, and 9 were used for immediate release. The dissolution rates from the FDM 3D-printed tablets could be increased by introducing pores and channels or lowering infill densities within tablets. Nevertheless, the dissolution rates remain relatively slow, which is due to the compact and non-disintegrating nature of printed tablets and the types of polymers and formulations used [81]. The same research group carried out a project to enhance drug release from FDM 3D-printed tablets involving the acid–base super solubilization technique, which means the interaction of poorly-water-soluble basic drugs with weak acids that would not form salts with the basic drug but would greatly increase its solubility [82].

### 3.7. PAT

PAT is used to design, analyze, and control processes and ensure product quality through timely measurements in a rapid and noninvasive way [83].

As is clear at this point of the review, HME and FDM are two separate techniques. Based on an article, the interruption of one process of HME-FDM disrupts the other and alters the CQA of a drug product. The use of PAT tools could enhance the robustness and efficiency of HME-FDM as a continuous manufacturing (CM) technique for the fabrication of high-quality customized drug products. Integration of PAT tools with QbD will determine the CQA for drug products. HME-FDM can be involved in CM with the application of PAT tools to provide real-time and in situ CQA monitoring and process control. Possible methods were found in the work of Bandari et al., where for the HME, in-line NIR or Raman spectroscopy could be implemented. Between the extruder and the FDM machine, a filament diameter detector and in-line texture imaging module could be added. For the FDM printing, at the print head an acoustic emission technique could be used for filament breakage monitoring, vibrational sensor-based monitoring could detect machine failure and product quality defects, and lastly heterogenous sensor-based monitoring could detect drifts in the printing process. As an in-line tool for the mapping of the ingredients, in-line NIR or Raman could be used, and for viscosity measurement an in-line rheometer could be used at the printer nozzle. In-line high-speed cameras could monitor filament surfaces as well, leading to better printability. The article also emphasizes the importance of the speed of the extrusion and 3D printing, which right now could be connected via an intermediate winding machine [23]. One research article emphasized that the solubility of a drug in a polymer matrix is a very important parameter that must be considered during polymer selection and offers different mathematical applications for the determination of that value. Also, the thermostability and the molecular weight have important roles, as the first parameter will affect the residence time and the latter will affect the viscosity, as high-molecular-weight polymers exhibit high melt viscosity [72].

The demand for continuous production and the exploration of PAT tools is not a new phenomenon. A research group assessed drug content as a critical quality attribute (CQA) using compact, low-cost NIR spectroscopy as a PAT tool. The critical parameters were the temperature of the FDM 3D printing and the drug concentration in the filament (10–15% *w*/*w*). The set model demonstrated that it has the ability to predict the concentration not only during the filament manufacturing but from 3D printed tablets with relatively low drug contents, small caplet designs, and complex formulas [41].

In an article, melt and flow behaviors were studied through CT scans, radiography, and force measurements. CT scans showed that higher filament speed increased the air gap between the filament and nozzle wall, while heater temperature had little effect. Radiography revealed a parabolic velocity profile, consistent with non-Newtonian flow simulations. These findings support more efficient nozzle design [84].

### 3.8. Guideline for QbD of HME-FDM

A study highlighted that 3D-printed products used in the medical field may be subject to the QbD approach in several aspects. It has the potential to control sources of variability and assist risk assessment in the FDM technique, while patient-based customization could also be included in the design space. The QbD approach can also facilitate the implementation of FDM 3D printing in pharmacy by using PAT, and it can also help with the acceptance of this new technology by regulatory authorities such as the European Medicines Agency (EMA) or FDA [85].

Even though the above-mentioned article highlights several important aspects of the future of 3D printing, the authors would like to draw their conclusions based on the information regarding HME-FDM discussed in this article.

As mentioned in Section 1 and Section 3.2.3, the input material attributes and the process parameters determining the quality attributes in HME are well described [18,23]. The authors claim that while there is not much more detailed information, some CMAs and CPPs could be added to the FDM portion of Section 3.3.3.

Based on all the examined literature, we would like to group the most important attributes into three categories: input material attributes (CMAs), HME equipment, and process parameters (CPPs); intermediate product analysis, FDM material parameters (CMAs), FDM equipment, and process parameters (CPPs); and final product attributes and monitoring methods. Table 5 and Table 6 summarize our recommendations for future HME-FDM projects, especially those aimed at drug delivery manufacturing. Our hope is that this guideline will give a real hand to those researchers who are interested in this area.

The opinion of the authors is that the CMAs and CPPs in hand with the performed analytical methods should be categorized into three categories: required tests, which must be performed for the proper product quality to be achieved; advisable tests, which should be performed due to the impact of the monitored parameter, but substantial analytical background is needed; and optional tests, whose effect on quality the authors are unable to assess based on the available information. In Table 5 and Table 6, the required tests are labeled with ++, the advisable with +, and the optional with Ø.

The authors would like to emphasize that in their opinion there are still some gaps regarding the available data, and thus proper guidelines are hard to give. The limitations of this review will be further discussed in Section 3.9.

Regarding the input material attributes, the thermal behavior and polymorph state description are required parameters. The particle size, distribution, and shape are advisable measurements to be performed, especially as pre-mixing of the powder mixture should be performed. The density and moisture content are not mentioned in the articles, so they might be optional.

The HME equipment parameters should be written more precisely with exact geometry data about screw elements and configurations, screw opening diameter, die diameter, and shape. The number of zones is also important and required information. If used, the feeder type and design should be recorded. If possible, the barrel volume might be stated. Measurement of these parameters requires no analytical background, and their reporting must be undertaken in all publications.

The CPPs of the HME process parameters are hard to give, as they may vary from formulation to formulation. For different drug delivery systems, numerous APIs and excipients are needed, and especially in the case of polymers, no universal range can be defined for most of the parameters. As a rule of thumb, the temperature range can be defined as 60–180 °C, and the screw speed as 30–100 rpm. Throughput is rarely quantified, and very few papers report SME, which limits reproducibility and cross-study comparison.

The required parameters are zone temperature, screw speed, and conveyor belt speed. The advisable values are barrel filling degree or specific feed loads (SFL), the SME, if applicable the solid and liquid feed rates, and environmental conditions (both temperature and humidity could be stated). As optional data, the cooling rate could be mentioned, but it is hard to interpret the data on the matter.

Intermediate product attributes such as the quality of the filament are very important and must be characterized by these methods: mechanical testing, filament diameter determination, FDM feedability and printability testing, physico-chemical characterization, and thermal behavior description. Rheology and particle size and shape determination might be advisable methods. These findings are in harmony with the FDM CMAs, as the mechanical flexibility must be tested, while rheology is advisable to perform.

The FDM equipment parameters can be described by the filament diameter, which is also an intermediate product attribute and highly recommended to test, as it affects the FDM feedability and printability. Platform leveling is required to be performed prior to each printing. The nozzle diameter is also required data, and according to the publications, 0.4 mm is the diameter that is most commonly used. The motor step size is optional information.

For the FDM process parameters, it is required to declare the printing and bed temperature, the printing speed, the layer height, and the infill percentage. The reviewed publications reported numerous other parameters, but there is no consistent description of which ones are absolutely necessary. The environmental conditions might be advisable to record.

The final product attributes are usually closely monitored by the researchers, as physico-chemical characterization, thermal analysis, microscopic characterization, and pharmacopeial dosage-form tests are mostly mentioned. All of these methods are in hand with the mechanical properties and the contact angle measurements. Furthermore, rheology might be an advisable measurement to be performed at this stage as well. Also, it is important to mention that biocompatibility tests should be performed, but they are not part of this article.

Taking all these factors into consideration, the authors believe that product quality can be improved and good reproducibility ensured, thereby eliminating batch-to-batch variability.

### 3.9. Limitations of the Review

A limitation of this work is the incomplete integration of QbD elements, including the explicit establishment of linkages between QTPP, CQAs, and CPPs/CMAs, as well as the definition of a design space. This primarily reflects the current state of the literature, as only a limited number of studies have systematically addressed these relationships in the context of HME-FDM manufacturing processes, especially in a continuous manufacturing process. Consequently, while this review identifies key parameters and explores their potential alignment with QbD principles, it does not provide a fully developed QbD framework. Instead, the findings highlight existing gaps and underscore the need for more structured, QbD-driven research to enable comprehensive integration in future studies.

Across HME-FDM studies, critical process information such as residence time, torque–process stability relationships, post-die filament cooling conditions, and real-time filament diameter monitoring is often omitted or only qualitatively described. Also, in the FDM process, there are still gaps on the software side, such as which printing parameters have the greatest impact or which nozzle should be used, and in-line techniques are rarely mentioned. There is no robust analytical testing scheme, as some methods are often but not always used, and some are rarely mentioned, despite their importance. This inconsistent reporting prevents meaningful cross-study comparison and data interpretation. As a result, despite the technological maturity of HME-FDM for pharmaceutical filament and drug delivery system manufacturing, these reporting gaps remain a major barrier to process standardization, scaleup, and regulatory translation.

## 4. Conclusions

HME-FDM is one of the most frequently published manufacturing methods within the 3D printing industry. Even though some articles mention the importance of the critical HME parameters and the required experiments to be performed, even recent publications barely perform tests regarding the material properties. Our aim is to emphasize the implementation of PAT tools and the QbD approach for the successful manufacturing of HME-FDM drug delivery systems, as they have the potential to scale up and be used as a continuous manufacturing technique. However, without the implementation of characterization methods, advanced batch processes cannot be achieved. In conclusion, we suggest the application of novel analytical and integration approaches to establish a resilient production system. We believe it would be crucial to determine quality by proper pre-analytical and analytical measurements for the persuasion of regulatory bodies worldwide that HME-FDM 3D-printed pharmaceuticals are safe and ready to be used. Suitable regulatory mechanisms should also be put in place to coordinate and harmonize HME-FDM as a continuous manufacturing strategy.

## Figures and Tables

**Table 1:** The input material attributes and the process parameters determining the quality attributes of HME products based on Yu et al. [18].

Input Material Attributes	Process Parameters	Quality Attributes
particle size and distribution fines/oversize particle shape melting point density solid form/polymorphic form moisture content	screw design, screw opening diameter screw speed solid and liquid feed rates feeder type/design number of zones zone temperatures chilling rate	extrudate density length thickness diameter polymorphic form and transition content uniformity throughput

**Table 2:** The grouping of the used hot-melt extrusion parameters. From the last decade, we chose 2–3 articles and filled in the table with four important information columns: the HME equipment details provided in the article, the used HME parameters, the used polymer type, and API. Used abbreviations: d = diameter, SME = specific mechanical energy input.

Year	HME Equipment Details	Used HME Parameters	Type of Polymer	Type of API	Article
2016	Counter-rotating, Twin-screw Die d = 1.8 mm (aluminum)	T = 160 °C Screw speed = 100 rpm Torque = 80 N·cm	Kollicoat^®^ IR (KIR)	Furosemide	[1]
		T = 65 °C Screw speed = 100 rpm Torque = 100 N·cm	PEO	None	
		T = 160 °C Screw speed = 70 rpm Torque = 70 N·cm	HPMC + 5% PEG 400		
		T = 165 °C Screw speed = 80 rpm Torque = 40 N·cm	HPC		
		T = 190 °C Screw speed = 70 rpm Torque = 80 N·cm	PVA		
		T = 120 °C Screw speed = 80 rpm Torque = 80 N·cm	Soluplus^®^ + 10% PEG 400		
		T = 180 °C Screw speed = 100 rpm Torque = 100 N·cm	HPMCAS + 5% PEG 8000		
		T = 160 °C Screw speed = 80 rpm Torque = 120 N·cm	Eudragit^®^ L		
		T = 120 °C Screw speed = 95 rpm Torque = 60 N·cm	Eudragit^®^ RL		
		T = 160 °C Screw speed = 100 rpm Torque = 100 N·cm	EC		
2016	Counter-rotating, Twin-screw, Die d = 1.5 mm	T = 100 °C Screw speed = 10 rpm	PCL	Indomethacin	[25]
2017	Co-rotating, Twin-screw, Die = 1 mm, Standard screw configuration	T = 180 °C in all zone Screw speed = 50 rpm	HPMC	Acetaminophen	[26]
		T = 140–160 °C in all zones Screw speed = 50 rpm	HPC LF or EF, EC, Soluplus^®^ or Eudragit^®^ L100		
2017	Co-rotating, Twin-screw, Die d = 1 mm	T = 80 °C Screw speed = 30 rpm	PEO, PLA, PVA	Rifampicin B	[27]
		T = 80 °C Screw speed = 10 rpm		Isoniazid	
2018	Not mentioned	T = 150 °C	Kollicoat^®^ IR	Aripiprazole	[28]
2018	Twin-screw, Die d = 1.75 mm	T = 100–120 °C Screw speed = 60 rpm Residence time = 10 min Torque = 40–160 N·cm	EC and release modifiers: HPMC, sodium alginate, PVA	Ibuprofen	[29]
2019	Die d = 2 mm, Standard screw configuration with three mixing zones	T = 100–155 °C Screw speed = 50 rpm	13 different polymers	Isoniazid	[30]
2019	Twin-screw, Standard screw configuration, Die d = 2 mm	T = depending on the formulation Screw speed = 50 rpm	AquaSolve™ HPMCAS LG and HG, Benecel™ HPMC E5 and K100M, Klucel™ HPC EF and HF, Aqualon™ EC N14	Acetaminophen	[31]
2020	Die d = 1.6 mm	T = 165 °C Screw speed = 35 rpm Cooling fan required	PVA	Diltiazem	[32]
		T = 180 °C Screw speed = 35 rpm	CA	None	
2020	Co-rotating, Twin-screw	T = 120 °C Screw speed = 40 rpm	PVP 40 Eudragit^®^ RSPO	Quercetin 1%	[33]
2020	Co-rotating, Twin-screw, Three mixing zones, Eight heating zones	T = 165 °C Screw speed = 50 rpm Torque = 400–500 N·cm	HPC and combination with EC	Theophylline	[34]
2021	Single-screw	T = 120–140 °C	PCL	Paracetamol	[35]
		T = 80–140 °C	PEO 200 K or PEO 100 K		
2021	Twin-screw, Seven heating zones, Die d = 2 mm, Detailed screw configuration	T_zone1_ = 75–90 °C T_zone2–7_ = 100–180 °C T_die_ = 130–180 °C Screw speed = 150–300 rpm Cooling by conveyor belt	Soluplus^®^, Kollidon^®^ VA64, and Eudragit^®^ E PO	Ketoconazole	[36]
2021	Twin-screw, Screw d = 10 mm, Screw L/D ratio 20	T_zone1_ = 80 °C T_zone2–4_ = 100 °C Screw speed = 60 rpm Winding machine	EC release modifier: PVA, Soluplus^®^, PEG 6000, Eudragit^®^ RL PO/RS PO, HPMC K4M/E10M/K100M, Kollidon^®^ VA 64/17PF/30	Ibuprofen	[24]
2022	Co-rotating, Twin-screw, Screw d = 12 mm, Screw L/D ratio 40, Detailed screw configuration with kneading zones, Die d = 2 mm, Gravimetric or volumetric feeder = 50 or 100 g/h	T_zone1–2_ = 20 °C T_zone3-die_ = 70–150 °C Screw speed = 25–50 rpm	Soluplus^®^ and Eudragit^®^ E PO	Enalapril maleate	[37,38]
2022	Experimental	T_feedport_ = 50 °C T = 160 °C Screw speed = 30 rpm	HPMC HME 15LV, PVP VA64, Eudragit^®^ EPO, Eudragit^®^ RS PO	Venlafaxine hydrochloride	[39]
2022	Co-rotating, Twin-screw, Screw d = 11 mm, Screw L/D ratio 40, Eight heating zones, Standard screw configuration, Manual feeding, Die round shaped, Die d = 2 mm and 2.5 mm	T = 145–160 °C Screw speed = 50 rpm Torque = 390–580 N·cm Cooling by conveyor belt	HPMC HME 15LV and 100LV, PEO, HPMCAS LG, MG and HG, PVA	Acetaminophen, Caffeine citrate	[40]
2023	Twin-screw	T = 110 °C	Eudragit^®^ EPO	Hydrocortisone	[41]
2023	Twin-screw, Screw d = 10 mm, Screw L/D ratio 20	T_feeding zone_ = 110 °C T_otherzones_ = 150 °C Screw speed = 50 rpm	HPC, EC	Theophylline	[42]
2023	Single-screw, Die d = 1.75 mm	T = 170–190 °C Screw speed = 65 rpm Feed rate = 60–120 g/h	PVA	Felodipine	[43]
2024	Co-rotating, Twin-screw, Die d = 2.7 mm	T_zone1_ = 90 °C T_zone2_ = 145 °C T_zone3_ = 150 °C T_zone4_ = 160 °C T_die_ = 170 °C Screw speed = 200 rpm Pressure, drive and SME also given Maximum torque = 1000 N·cm Feed rate = 300 g/h	HPMC	Theophylline	[44]
2024	Co-rotating, Twin-screw, Screw d = 11 mm, Screw L/D ratio 40, Eight heating zones, Die d = 2.5 mm, Manual feeding, Conveyor belt	T = 160–170 °C Screw speed = 50 rpm	Affinisol™ HPMC HME 15LV, Parteck^®^ MXP PVA, EMPROVE^®^ ESSENTIAL, Kollicoat^®^ IR, and VA64	Caffeine	[45]
2024	Co-rotating, Twin-screw, Screw d = 11 mm, Screw L/D ratio 40, Eight heating zones, Die = 1.5 mm, Standard screw configuration with three mixing zones	T = 160 °C Screw speed = 50 rpm	Parteck^®^ MXP PVA 3–82	Acetaminophen	[46]
2025	Co-rotating, Twin-screw, Die d = 1.75 mm, Gravimetric and force feeder, Conveyor belt	T = 70 °C Screw speed = 50 rpm	PEO	Sodium valproate	[13]
2025	Die d = 1.75 mm	T = 60–70 °C Screw speed = 30 rpm	PEO	Propranolol hydrochloride	[47]
2025	Co-rotating, Twin-screw, Screw d = 12 mm, Die d = 1.75 mm Eight heating zones, Detailed screw configuration	T_1_ = 40 °C T_2_ = 50 °C T_3_ = 60 °C T_4–7_ = 80 °C T_8_ = 70 °C T_9_ = 60 °C Screw speed = 50 rpm	PCL MW50000	Gabapentin	[48]

**Table 3:** The published material, machine, and process parameters of the products in the above-mentioned articles. Used abbreviations: APAP—acetaminophen; CC—caffeine citrate; ENP—enalapril maleate; DKP—enalapril diketopiperazine; IBP—ibuprofen; ISO—isoniazid; KTZ—ketoconazole; PBO—placebo; RIF—rifampicin B, TEO—theophylline; SA—sodium alginate; TEC—triethyl citrate; VA-64—vinylpyrrolidone-vinyl acetate copolymer; XG—xanthan gum.

Year	Material Parameters	Machine Parameters	Process Parameters	Article
2016	EC; GLY; KIR; HPC; HPMC; PEO; PEG 400, PEG 8000; PVA; SLP; TEC; Eudragit^®^ L, RL	MakerBot^®^ Replicator 2X (MakerBot Industries, Brooklyn, NY, USA) Nozzle = 0.4 mm Modified feeding mechanism Unheated build plate	T_print_ = 160–225 °C Infill = 100% Layer height = 0.3 mm	[1]
2016	PCL + Indomethacin	MakerBot^®^ Replicator 2X Build plate material = Kapton^®^	T_loading_ = 120 °C T_printing_ = 100 °C Printing speed = 45 mm/s Travel speed = 150 mm/s Infill = 10% Layer height = 0.1 mm Number of shells = 3	[25]
2017	HPC; HPMC; Eudragit^®^ L100; Soluplus^®^; APAP	Prusa i3 E3D V6 hot end (Prusa Research, Prague, Czech Republic) Nozzle = 0.4 mm	T_print_ = 200 °C T_bed_ = 50 °C Printing speed = 50 mm/s Travel speed = 50 mm/s Infill = 100% Layer height = 0.1 mm Outside shell thickness = 0.4 mm	[26]
2017	PEO; PLA; PVA + RIF or ISO	Ultimaker 3 Extended dual-nozzle printer (Ultimaker, Utrecht, The Netherlands) Nozzle = 0.4 mm	T_print_ = 210–225 °C T_bed_ = 60 °C Printing speed = 35 mm/s Infill = 100% Layer height = 0.2 mm	[27]
2018	PLA + Kollicoat^®^ IR + Aripiprazole	ZMorph^®^ 2.0 SX DualPro extruder (ZMorph S.A., Wrocław, Poland) Nozzle = 0.4 mm	T_print_ = 208–210 °C T_bed_ = 60 °C Printing speed = 8–10 mm/s Layer height = 0.2 mm Path width = 0.4 mm	[28]
2018	EC; HPMC; PVA; SA; XG; IBP API% = 16–24%	JG Aurora A3 (JG Maker, Shenzhen, China) Nozzle = 0.4 mm	T_print_ = 174–182 °C Printing speed = 7.5–75 mm/s Travel speed = 100 mm/s Infill = 15–25% Layer height = 0.1–0.3 mm Shell thickness = 0.4–1.2 mm Minimal layer time for cooling = 5 s	[29]
2019	HPC; HPMC; PEO; PLA; TEC; Eudragit^®^ RS PO, RL PO, L 100; Kolliphor^®^ TPGS; ISO	MakerBot Replicator 2X Custom-built air-cooled print head Build plate material = glass with blue tape	T_print_ = 165–195 °C Printing speed = 90 mm/s Travel speed = 150 mm/s Infill = 15%, 90% Layer height = 0.05 mm Outlines printed on each layer = 2	[30]
2019	EC; HPC; HPMC; HPMCAS; APAP	Prusa i3 E3D V6 hot end Nozzle = 0.4 mm	T_print_ = 200 °C T_bed_ = 50 °C Printing speed = 50 mm/s Travel speed = 50 mm/s Infill = 100% Layer height = 0.1 mm Outside shell thickness = 0.4 mm	[31]
2020	PVA + PVP K30 + Diltiazem HCl + Mannitol	MakerBot Replicator 2X	T_print_ = 205–215 °C T_bed_ = 110 °C Printing speed = 20 mm/s First layer printing speed = 7 mm/s Travel speed = 50 mm/s Infill = 100% Layer height = 0.2 mm Number of shells = 2 Floor thickness = 0 mm Roof thickness = 1.2 mm	[32]
2020	TEC + PVP-40 + Eudragit^®^ RS PO + Quercetin	Ultimaker 3 (Ultimaker, Utrecht, The Netherlands) Nozzle = 0.4 mm Building plate material = flexible polyester backing membrane	T_print_ = 200 °C T_bed_ = 40 °C Printing speed = 50 mm/s Infill = 100% Layer height = 0.1 mm Fan speed = 70%	[33]
2020	EC; HPC	Prusa i3 E3D V6 hot end Nozzle = 0.4 mm	T_print_ = 190 °C T_bed_ = 60 °C Printing speed = 50 mm/s Travel speed = 50 mm/s Infill = 100% Layer height = 0.1 mm Shell thickness = 0.8–2.0 mm Wall thickness = 0.0–1.6 mm/s	[34]
2021	PCL; PEO; APAP	Ultimaker 3 Nozzle = 0.8 mm	T_print_ = 140–170 °C T_bed_ = 60–80 °C Infill = 70%	[35]
2021	SLP; VA-64; Eudragit^®^ E PO; PBO; KTZ	Ultimaker 3 Modified for 1.75 mm filaments Bowden extruder Nozzle = 0.4 mm	T_print_ = 140–190 °C T_bed_ = 20–70 °C Printing speed = 10–30 mm/s Infill = 100% Layer height = 0.2 mm Line width = 0.4 mm	[36]
2021	EC; HPMC K4M, E10M, K100M; PEG 6000; PVA; VA-64; TEC; Eudragit^®^ RL PO, RS PO; Kollidon^®^ 17 PF, 30; Soluplus^®^	MakerBot Replicator 2X MK8 dual-head extruder Nozzle = 0.4 mm	T_print_ = 178 °C T_bed_ = 65 °C Infill = 100% Layer height = 0.2 mm	[24]
2022	PEO; Eudragit^®^ E PO; Soluplus^®^; ENP; DKP	Prusa i3 Mk3 (Prusa Research, Prague, Czech Republic) Nozzle = 0.4 mm, 0.6 mm	T_print_ = 180 °C, 190 °C T_bed_ = 35 °C Printing speed = 30 mm/s, 60 mm/s, 90 mm/s Infill = 100% Layer height = 0.2 mm	[37,38]
2022	PLA (left nozzle) Drug-loaded filaments (right nozzle): venlafaxine HCl; HPMC; TECO; VA-64 Eudragit^®^ E PO, RS PO	Raise 3D Pro-2 series (Raise3D Technologies, Inc., Irvine, CA, USA) Dual-nozzle Nozzle = 0.4 mm	T_print(left)_ = 210 °C T_print(right)_ = 185 °C T_bed_ = 60 °C Infill = 100% Layer height = 0.2 mm Line width = 0.4 mm	[39]
2022	HPMC; HPMCAS; PEO; PLA; PVA; APAP; CC Filament diameter = 2.6–2.85 mm	Ultimaker 3 Dual-nozzle Bowden extruder	T_print_ = 190–215 °C T_bed_ = 50 °C Printing speed = 50 mm/s Infill = 100% Layer height = 0.1 mm	[40]
2023	HC + Eudragit^®^ E PO + TiO_2_ + Talc + Sodium stearyl fumarate + TEC	MakerBot Replicator 2X	T_print_ = 140 °C T_bed_ = 60 °C Printing speed = 25 mm/s Travel speed = 20 mm/s Infill = 100% Layer height = 0.2 mm	[41]
2023	30% TEO + 35% HPC JF + 35%% EC 30% TEO + 70% HPC EF	Not mentioned	T_print_ = 220 °C T_bed_ = 50 °C Printing speed = 90 mm/s Infill = 100% Layer height = 0.2 mm Number of shells = 2	[42]
2023	5% Felodipine + 5% Mannitol + 90% PVA	MakerBot Replicator 2X	T_print_ = 165 °C Infill = 10%, 50%, 80%	[43]
	15% Felodipine + 5% Mannitol + 80% PVA	Not mentioned	T_print_ = 180 °C Infill = 10%, 50%, 80%	
2024	Not mentioned	Not mentioned	Not mentioned	[44]
2024	HPMC; KIR; PVA; VA-64; Sorbitol; Caffeine Filament diameter = 2.6–2.85 mm	Ultimaker 3 Dual-nozzle Bowden extruder Nozzle = 0.4 mm	T_print_ = 175–195 °C T_bed_ = 50 °C Printing speed = 50 mm/s Infill = 100% Layer height = 0.1 mm	[45]
2024	85% PVA + 15% APAP	Prusa i3 Mk3 Nozzle = 0.4 mm	T_print_ = 180 °C T_bed_ = 50 °C Printing speed = 50 mm/s Infill = 100% Infill pattern = lines Layer height = 0.15 mm Line width = 0.4 mm No extra top or bottom layers	[46]
2025	PEG 6K; PEG 35K; PEO; Sodium valproate	Raise 3D Pro-2 Nozzle = 0.4 mm	T_print_ = 100–140 °C Printing speed = 5 mm/min Infill = 50% Layer height = 0.5 mm	[13]
2025	PEO + PEG 6000 + Propranolol HCl	Prusa i3 Mk3 Nozzle = 0.4 mm	First layer T_print_ = 190 °C First layer T_bed_ = 50 °C Second layer T_print_ = 180 °C Second layer T_bed_ = 45 °C Printing speed = 30 mm/s Infill = 100% Infill pattern = rectilinear Layer height = 0.15 mm Number of shells = 2	[47]
2025	PCL + PEG 3350 + Gabapentin	MakerBot Replicator 2X Nozzle = 0.4 mm	T_print_ = 110 °C T_bed_ = 30 °C Printing speed = 5 mm/s Infill = 25%, 50%, 100% Infill pattern = linear Layer height = 2 mm	[48]

**Table 4:** The performed pre-analytical, intermediate, and final product characterization tests (except for the pharmacopeial dosage-form tests, e.g., dissolution test, uniform mass) of the products in the selected above-mentioned articles.

Year	Pre-Analytical Test	Intermediate Product Analytical Test	Final Product Analytical Test	Article
2016	Some materials were kept in an oven at 40 °C for 24 h prior to use.	Not mentioned.	Not mentioned.	[1]
2016	Raw material PXRD and DSC	PXRD, DSC, SEM, Rheology	PXRD, DSC, SEM	[25]
2017	Raw material DSC and TGA	DSC and TGA, 3PB	DSC and TGA	[26]
2017	Raw material DSC and TGA	PXRD, DSC and TGA	PXRD, DSC and TGA, SEM	[27]
2018	Not mentioned	Stereoscopic microscope and PLM	PXRD, Stereoscopic microscope and PLM	[28]
2018	Viscosity of EC was mentioned	DSC and TGA, Stereoscopic microscope, Tensile and hardness test	DSC and TGA, Stereoscopic microscope	[29]
2019	Raw material DSC	DSC, Dynamic vapor sorption analysis (DVSA), 3PB	DSC	[30]
2019	DSC and TGA, PLM, Rheology by shear rheometer	PXRD, FTIR, Repka–Zhang test, Rheology by shear rheometer In-line NIR	SEM	[31]
2020	Raw material PXRD, DSC and TGA	PXRD, DSC and TGA, SEM, optical microscopy and elemental analysis studies, Mechanical tests: instrumented indentation testing (IIT), tensile tests	PXRD, DSC and TGA, SEM, optical microscopy and elemental analysis studies, XRCT,	[32]
2020	Raw material PXRD and DSC	In-line diameter determination, PXRD, DSC	PXRD, DSC, SEM, Moisture content	[33]
2020	TGA	SEM, Repka-Zhang test	SEM	[34]
2021	Raw material PXRD and DSC	PXRD, DSC, 3BP	PXRD, DSC,	[35]
2021	Not mentioned	PXRD, DSC, PLM, 3BP	PXRD, DSC, PLM	[36]
2021	Raw materials DSC and TGA	PXRD, DSC, SEM, Mechanical characterization of filament	PXRD, DSC, SEM,	[24]
2022	Powder mixture determination: Particle size, DSC and derivative TGA (DTGA), FTIR SEM	PXRD, DSC and DTGA SEM	PXRD, DSC and DTGA, SEM	[37,38]
2022	Raw materials: PXRD, DSC and TGA, FTIR	PXRD, DSC and TGA, FTIR	PXRD, DSC and TGA, FTIR	[39]
2022	PXRD, DSC	3PB, resistance, and stiffness test	SEM	[40]
2023	Raw materials: PXRD, DSC and TGA	PXRD, DSC and TGA	PXRD and NIR, DSC and TGA, SEM	[41]
2023	Not mentioned.	Not mentioned.	Not mentioned.	[42]
2023	DSC for the physical mixtures	DSC, 3BP, Repka-Zhang, and stiffness test	Not mentioned.	[43]
2024	Raw material particle size and particle size distribution, Melt viscosity by rotary rheometer	Scanning Raman microscopy for particle analysis in the filament, 3PB test	Not mentioned.	[44]
2024	DSC	PXRD and FTIR, DSC	PXRD and FTIR, DSC	[45]
2024	DSC	FTIR, DSC, 3PB test	FTIR, DSC, SEM	[46]
2025	PXRD, DSC and TGA	PXRD, DSC and TGA, SEM, 3PB test, Rheology: small amplitude oscillatory shear (SAOS) measurements	PXRD, DSC and TGA	[13]
2025	PXRD, DSC and TGA	PXRD, DSC and TGA, Microscopic imaging by Keyence digital microscope, 3PB test	Microscopic imaging by Keyence digital microscope and focus variation microscopy, contact angle measurement	[47]
2025	PXRD, DSC, Vacuum compression modeling (VCM)	PXRD, DSC, SEM and Energy-Dispersive X-ray (EDX) Spectroscopy, Repka–Zhang-test	PXRD, DSC, Optical microscopy, SEM, and EDX	[48]

**Table 5:** Recommendations on critical parameters of HME and possible methods for monitoring them (labeled by italics). The priority of each parameter is labelled as required “**++**”, advisable “**+**”, or optional “**Ø**”.

Input Material Attributes—CMAs	HME Equipment Parameters—CPPs	HME Process Parameters—CPPs	Intermediate Product Attributes—CMAs
Particle size and distribution characterization (*laser diffraction, sieving, DLS*) **+** Particle shape classification, homogeneity check (*SEM or optical microscopy or dynamic image analysis, automated static image analysis*) **+** Thermal behavior description (*DSC, TGA*) **++** Polymorph state description (*PXRD*) **++** Density (*gas pycnometer*) Ø Moisture content (*moisture analyzer, Karl Fischer titration, TGA, moisture sensor of NIR*) Ø	Geometry: screw elements and configuration, screw opening diameter, die diameter and shape **++** Number of zones **++** Feeder type/design **++** Barrel volume **+**	Zone temperatures **++** Screw speed **++** Barrel filling degree or specific feed loads (SFL) **+** SME **+** Solid and liquid feed rates (powder feed rate—PFR) **+** Cooling rate Ø Conveyor belt speed **++** Environment conditions: temperature and humidity **+**	Mechanical testing (*texture analyzer: 3BP, Repka–Zhang test, resistance test, stiffness test*) **++** Rheology (*capillary or oscillatory rheometers, XPTV*) **+** Filament diameter (*caliper, laser-based module*) **++** FDM feedability and printability (*special method by texture analyzer, actual dosing into the FDM printer*) **++** Physico-chemical characterization (*PXRD, FTIR, NIR, NMR*, *scanning Raman microscopy*, *gas pycnometer*) **++** Thermal behavior description (*DSC, TGA*) **++** Particle size, fines/oversize, and shape determination (*laser diffraction, DLS, SEM or optical microscopy or dynamic image analysis, automated static image analysis*) Ø

**Table 6:** Recommendations on critical parameters of FDM and possible methods for monitoring them (labeled by italics). The priority of each parameter is labelled as required “**++**”, advisable “**+**”, or optional “**Ø**”.

FDM Material Parameters—CMAs	FDM Equipment Parameters—CPPs	FDM Process Parameters—CPPs	Final Product Attributes and Monitoring Methods
Melting rheology **+** Melting flexibility **++**	Geometry (e.g., *nozzle diameter*) **++** Filament diameter **++** Motor step size Ø Platform leveling **++**	Temperature (*nozzle, platform*) **++** Printing speed **++** Layer thickness **++** Infill percentage **++** Other Printing parameters (e.g., extrusion width, shell thickness) **+** Environment conditions: temperature and humidity **+**	Physico-chemical characterization (*PXRD, FTIR, NIR, NMR*) **++** Thermal analysis (*DSC, TGA*) **++** Microscopic characterization (*SEM, micro-CT, HSM, PLM, goniometer*) **++** Pharmacopeial dosage-form specific tests **++** Mechanical properties (*texture analysis, physical stress tests*) **++** Rheology (*capillary or oscillatory rheometers, XPTV*) **+** Contact angle measurement **++**

## Data Availability

The original contributions presented in this study are included in the article/Supplementary Materials. Further inquiries can be directed to the corresponding author.

## Supplementary Materials

The following supporting information can be downloaded at: https://www.mdpi.com/article/10.3390/pharmaceutics18050569/s1, PRISMA 2020 Checklist. Ref. [86] cited in Supplementary Materials.

## Abbreviations

The following abbreviations are used in this manuscript:

QbD	quality by design
HME-FDM	hot-melt extrusion coupled fused deposition modeling
HME	hot-melt extrusion
FDM	fused deposition modeling
API	active pharmaceutical ingredient
QTPP	quality target product profile
CMA	critical material attribute
CQA	critical quality attribute
PLA	polylactic acid
PVA	polyvinyl alcohol
PCL	polycaprolactone
ABS	acrylonitrile butadiene styrene
HIPS	high impact polystyrene
PVP	polyvinylpyrrolidone
EC	ethylcellulose
HPC	hydroxypropylcellulose
HPMC	hydroxypropyl methylcellulose
HPMCAS	hydroxypropyl methylcellulose acetate succinate
PEO	polyethylene oxide
EVA	ethylene vinyl acetate
FDA	U.S. Food and Drug Administration
L/D	length-to-diameter
CPPs	critical process parameters
KIR	Kollicoat^®^ IR
CA	cellulose acetate
DSC	differential scanning calorimetry
PXRD	powder X-ray diffraction
APAP	acetaminophen
CC	caffeine citrate
ENP	enalapril maleate
DKP	enalapril diketopiperazine
IBP	ibuprofen
ISO	isoniazid
KTZ	ketoconazole
PBO	placebo
RIF	rifampicin B
TEO	theophylline
SA	sodium alginate
TEC	triethyl citrate
VA-64	vinylpyrrolidone-vinyl acetate copolymer;
XG	xanthan gum
DLS	dynamic light scattering
SEM	scanning electron microscopy
TGA	thermogravimetric analysis
NIR	near-infrared spectroscopy
FTIR	Fourier transform infrared spectroscopy
NMR	nuclear magnetic resonance spectroscopy
micro-CT or µCT	micro-computed tomography
XRCT	X-ray computed tomography
HSM	hot-stage microscopy
PLM	polarized light microscopy
XPTV	X-ray particle tracking velocimetry
DVSA	dynamic vapor sorption analysis
IIT	instrumented indentation testing
DTGA	DSC and derivative TGA
SAOS	small-amplitude oscillatory shear
EDX	energy-dispersive X-ray spectroscopy
Tm	melting temperature
Tg	glass transition temperature
Td	degradation temperature
PFR	powder feed rate
SFL	specific feed loads
VCM	vacuum compression modeling
CM	continuous manufacturing
EMA	European Medicines Agency
